# Disentangling the effects of phonation and articulation: Hemispheric asymmetries in the auditory N1m response of the human brain

**DOI:** 10.1186/1471-2202-6-62

**Published:** 2005-10-15

**Authors:** Hannu Tiitinen, Anna Mari Mäkelä, Ville Mäkinen, Patrick JC May, Paavo Alku

**Affiliations:** 1 Apperception & Cortical Dynamics (ACD), Department of Psychology, P.O.B. 9, FIN-00014 University of Helsinki, Finland; 2 BioMag Laboratory, Engineering Centre, Helsinki University Central Hospital, Finland; 3 Laboratory of Acoustics and Audio Signal Processing, Helsinki University of Technology, Espoo, Finland

## Abstract

**Background:**

The cortical activity underlying the perception of vowel identity has typically been addressed by manipulating the first and second formant frequency (F1 & F2) of the speech stimuli. These two values, originating from articulation, are already sufficient for the phonetic characterization of vowel category. In the present study, we investigated how the spectral cues caused by articulation are reflected in cortical speech processing when combined with phonation, the other major part of speech production manifested as the fundamental frequency (F0) and its harmonic integer multiples. To study the combined effects of articulation and phonation we presented vowels with either high (/a/) or low (/u/) formant frequencies which were driven by three different types of excitation: a natural periodic pulseform reflecting the vibration of the vocal folds, an aperiodic noise excitation, or a tonal waveform. The auditory N1m response was recorded with whole-head magnetoencephalography (MEG) from ten human subjects in order to resolve whether brain events reflecting articulation and phonation are specific to the left or right hemisphere of the human brain.

**Results:**

The N1m responses for the six stimulus types displayed a considerable dynamic range of 115–135 ms, and were elicited faster (~10 ms) by the high-formant /a/ than by the low-formant /u/, indicating an effect of articulation. While excitation type had no effect on the latency of the right-hemispheric N1m, the left-hemispheric N1m elicited by the tonally excited /a/ was some 10 ms earlier than that elicited by the periodic and the aperiodic excitation. The amplitude of the N1m in both hemispheres was systematically stronger to stimulation with natural periodic excitation. Also, stimulus type had a marked (up to 7 mm) effect on the source location of the N1m, with periodic excitation resulting in more anterior sources than aperiodic and tonal excitation.

**Conclusion:**

The auditory brain areas of the two hemispheres exhibit differential tuning to natural speech signals, observable already in the passive recording condition. The variations in the latency and strength of the auditory N1m response can be traced back to the spectral structure of the stimuli. More specifically, the combined effects of the harmonic comb structure originating from the natural voice excitation caused by the fluctuating vocal folds and the location of the formant frequencies originating from the vocal tract leads to asymmetric behaviour of the left and right hemisphere.

## Background

A voiced speech signal such as a vowel is created in the human sound production system through phonation and articulation [1]. In normal phonation, the vibrating vocal folds produce a periodic excitation, termed the glottal flow. Due to this inherent periodicity, the spectra of vowels produced by normal phonation are characterized by a harmonic comb structure, i.e., distribution of energy at the fundamental frequency (F0, ranging from 100 Hz in males up to 400 Hz in infants) and its harmonic integer multiples (2 × F0, 3 × F0, etc.) located regularly in frequency [2]. This comb structure is then locally weighted in frequency by the resonances caused by the vocal tract. These resonances, termed the formants (F1, F2, F3, etc.), determine the vowel category. Changing the shape and the length of the vocal tract results in different formant frequency settings and, consequently, in variations of the perceived phoneme category. The F0 and its harmonics are the primary acoustical cues underlying pitch perception and the lowest two formants are regarded as the major cues in vowel categorization [1].

The auditory N1(m) response of the electro- and magnetoencephalography (EEG & MEG, respectively), generated in the auditory cortices of the left and right hemisphere, reflects the acoustic properties of auditory stimuli [[3-10], see [11] for a review]: its amplitude is largely determined by stimulus onset characteristics and stimulus intensity and its latency varies according to both stimulus intensity and frequency. An increase in stimulus intensity decreases the latency of the N1m and, in the 500 – 4000 Hz range, the N1m is elicited at a roughly invariant latency. Interestingly, in the frequency range of speech F0, sinusoidal stimuli result in longer-latency N1(m) responses and this latency delay increases monotonically as stimulus frequency is lowered [12,13].

With respect to phonation, the latency delay of the N1m is observable both when the F0 is present [14] and absent [11,15,16]; in the latter case, provided that the harmonic structure of the high-frequency components is intact, the result is the virtual perception of the fundamental frequency (i.e., the missing fundamental). With regard to articulation, the categorization of vowels might be based on temporal encoding of the formant frequencies [6,7,17,18]. For instance, the vowel /u/, which has relatively low F1 and F2 values (approx. 300 & 800 Hz, respectively), elicits the N1(m) at a longer latency than the vowel /a/, which has higher F1 and F2 values (700 & 1100 Hz, respectively). Previous studies have related these effects either to the F1 [11,18] or F1 and F2 values [6,7,17] of these vowels.

These latency effects of the N1m elicited by vowels have been documented to occur symmetrically in the two hemispheres [6,7,11,17,18]. This symmetry appears rather interesting when considering that speech stimuli comprising consonants [4,19] have been found to elicit asymmetric N1m response behavior. However, given that vowels are the core phonemes of speech utterances [2], and that they comprise spectral energy preferred by either the left or the right hemisphere (i.e., formant frequencies and glottal periodicity, respectively; [20]), one would expect that isolated vowel sounds should result in hemispheric asymmetries as indexed by the auditory N1m response. Hemispheric specificity of speech processing notwithstanding, no consensus has been reached on whether cerebral asymmetries are brought about only by attentional top-down modulation of cortical activity [21] or whether they might be found already in the passive recording condition when the subject is not engaged in the attentive processing of vowel stimuli.

To summarize, the effects of voice excitation and articulation on cortical activity elicited by vowels have been studied extensively – but, more often than not, in isolation. This, obviously, might be considered a shortcoming in cognitive brain research, further emphasized by the fact that the two issues are inseparable in real speech communication. In addition, studies addressing the combined effects of phonation and articulation have typically used a much too narrow perspective in characterizing voice excitation; it is often quantified in terms of F0 alone while the role of the *type *of the excitation, and thereby also the set of underlying spectral cues, is ignored. This limited perspective, again, can be criticized from the point of view of natural speech communication: As an example, two representatives of the vowel /a/ can be created with equal F0s but with greatly different types of the voice excitation waveform. This results in two speech sounds, both perceived as the phoneme /a/ and, importantly, of the same pitch. However, their voice quality can be clearly different due to differences in the type of the excitation waveform. It is, for example, possible that the one /a/ sounds breathy due to use of a soft pulseform in the glottal excitation whereas the voice quality of the other /a/ is perceived as pressed resulting from the use of a sharper shape in the glottal excitation pulseform [22,23].

Besides the above-mentioned, restricted view on the role of the voice excitation type, we hasten to emphasize another, equally overlooked an issue in studies of speech production and perception: because of the wide range of their F1 and F2 values, vowels are also fundamentally different in terms of the distribution of energy over frequency. For instance, due to its high F1 and F2, the sound energy in the vowel /a/ is distributed across a wide, 0–2 kHz range of high-energy harmonics. However, in the case of, say, vowel /u/, the low positions of F1 and F2 strongly attenuate the higher harmonics and most of the sound energy is actually allocated at frequencies below 1 kHz. This, then, results in variations in the perceived loudness of the stimuli, despite attempts to adjust the intensity of the stimuli using objective measures such as the sound pressure level (SPL).

Recent studies conducted in the passive recording condition indicate that the overall harmonic structure of vowels should perhaps not be overlooked in descriptions of speech-evoked cortical activity. For one, the amplitude of the N1m is already modulated by the presence of periodic glottal excitation in vowel sounds: a vowel with this kind of excitation elicits larger-amplitude N1m responses than the same vowel with an aperiodic, intensity-matched noise excitation [24]. Further, the amplitude of the N1m reflects temporal changes in the harmonic structure of speech created by glides in F0 while corresponding glides in pure tones do not affect the N1m amplitude [25]. Contrasting these observations, both the amplitude and latency of the N1m are unaffected by the identity of loudness-matched vowels (/a/, /o/, & /u/) [26] and by the lack of phonetic F1,F2-content in natural, periodically excited vowels [27]. Regardless of the formant frequencies, the latency of the N1m elicited by speech sounds with different F0-values appears to be invariant and shorter than the latency of the N1m elicited by pure tones whose frequencies are adjusted to match the F0 of the speech sounds [25,27]. Thus, these findings tentatively suggest that the presence of periodic glottal excitation in auditory stimulation might be an important prerequisite for the elicitation of speech-specific cortical activity.

**Figure 1:**
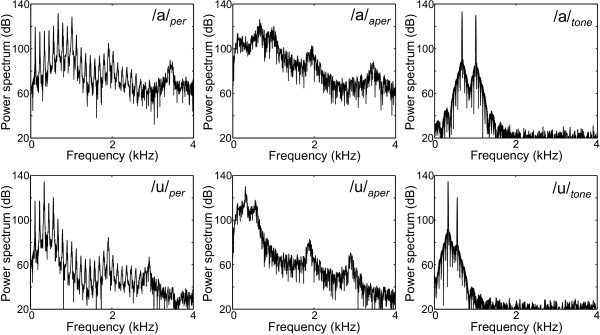
The spectra of the stimuli for the vowels /a/ (upper row) and /u/ (lower row), representing how articulation modifies stimulus structure. The stimuli were created using three different types of phonation: the natural periodic glottal pulseform (sounds /a/_*per *_and /u/_*per *_in the left column), the aperiodic noise sequence (/a/_*aper *_and /u/_*aper*_, center column), and tonal excitation (/a/_*tone *_and /u/_*tone*_, right column). The vowels excited by the natural periodic glottal pulseform are characterized by a harmonic comb structure, that is, distribution of sound energy at multiple integers of the fundamental frequency. This regular spectral fine structure is absent from the spectra of the vowels produced by the aperiodic excitation. The spectra of the sounds generated by tonal excitation are further impoverished, comprising only two spectral components. The spectral characteristics of the stimuli of all three excitation types are affected by the formant structure of the underlying vowel. Due to this, the vowel /a/ comprises more high frequencies than the vowel /u/.

Given the lack of data on the combined effects of phonation and articulation, the present study was designed to investigate how different combinations of voice excitation (phonation) and formant frequencies (articulation; for a description of the stimuli, see Fig. 1) are reflected in the cortical processing of vowels as indexed by the auditory N1m response. To investigate the effects of phonation, we used the periodic glottal excitation extracted from a natural utterance and contrasted its effects with those of an aperiodic noise waveform and a tonal excitation represented by two sinusoids. The effects of articulation, in turn, were analyzed by introducing two natural-sounding vowels with an intact harmonic structure (/a/_*per *_& /u/_*per*_) and located in the opposite corners of the F1,F2-space. Hence, as illustrated in Fig. 1, the study comprised two phonemes with known formant values, but created by three substantially different variants of excitation. The spectra of the vowels excited by aperiodic noise (/a/_*aper *_& /u/_*aper*_) were similar to their periodic counterparts, both in terms of the formant frequencies and the overall spectral envelope structure but, importantly, they lacked the comb structure of natural speech. Further impoverishing the stimulation, we also utilized two-tone complexes /a/_*tone *_and /u/_*tone*_, where the sound energy was concentrated at two distinct frequency peaks corresponding to the F1 and F2 of /a/ and /u/.

Perceptually, the vowels /a/_*per *_and /u/_*per *_were of normal voice quality while their aperiodic, noise-excited counterparts matched for intensity resembled whispered speech. Both had a rich spectral structure and were recognizable as speech. In contrast, the tonal stimuli had an extremely sparse spectral structure not perceivable as speech. Based on previous research [11,12,14-16,24-27], we hypothesized that the type of phonation (voice excitation) should be reflected in latency variations of the N1m response. With regard to articulation, we expected that the different sound energy distributions of the vowels /a/ and /u/, caused by the different articulatory settings as explained above, should result in variations in the amplitude of the N1m. With regard to amplitude, latency, and source localization of the N1m, we were specifically interested to see whether asymmetries in the left- vs. right-hemispheric brain activity might arise already in the passive recording condition. Finally, in line with the tentative findings reported in [24], the experimental design allowed us to study whether human speech consisting of an intact, natural harmonic structure leads to a different spatial distribution of cortical activation than unnatural utterances.

## Results

**Figure 2:**
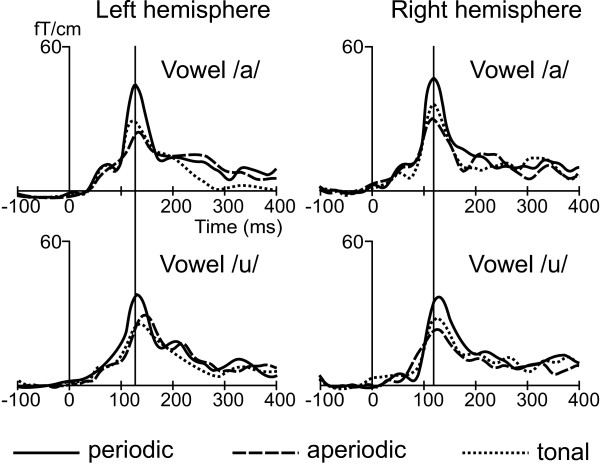
Grand-averaged (*N *= 10) responses elicited by periodic (thick line), aperiodic (dashed line), and tonal (dotted line) excitation of the vowel /a/ and /u/, calculated over the pair of planar gradiometers displaying N1m response maxima above the left and right hemisphere. In all cases, the stimuli comprising natural periodic structure elicited a prominent N1m response peaking at around 120 ms after stimulus onset.

**Figure 3:**
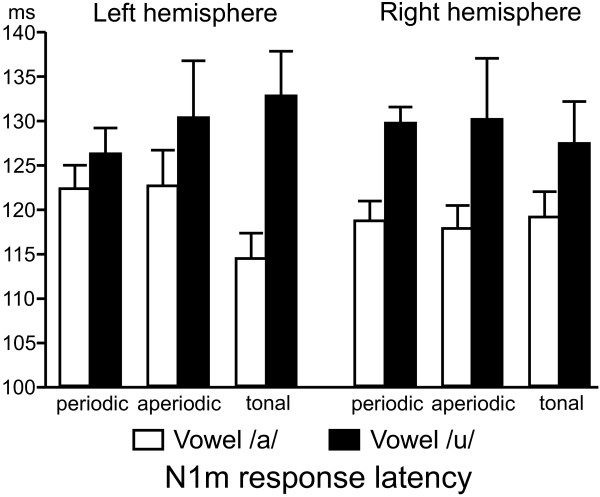
The grand-averaged latency of the left- and right-hemispheric N1m for the vowels /a/ and /u/ with three different types of phonation (periodic, aperiodic & tonal). In both hemispheres, the N1m for the vowel /a/ was elicited, on the average, 10 ms earlier than that for /u/. The latency behavior of the N1m was asymmetric across the two hemispheres: In the right hemisphere, N1m latency was determined by articulation (vowel category), whereas the latency of the left-hemispheric N1m depends on both phonation and articulation. Notably, in the left hemisphere, there were no significant latency differences between the N1m responses elicited by the periodic vowels /a/_*per *_and /u/_*per*_. Bars indicate standard error of the mean.

As illustrated in Figures 2 and 3, the temporal dynamics of cortical activation as indexed by the latency of the N1m varied asymmetrically in the right and left hemispheres according to vowel category and type of excitation. This observation was confirmed by statistical analysis which showed a significant hemisphere by vowel by excitation type-interaction (*F*(2,18) = 9.55, *p *< 0.01): In the right hemisphere, the periodic, aperiodic, and tonal variants of /a/ elicited the N1m at an invariant latency (119, 118, and 119 ms for /a/_*per*_, /a/_*aper*_, and /a/_*tone*_, respectively; *p *= n.s. in all comparisons), and, interestingly, some 10 ms earlier than the three variants of /u/ (130, 130, and 127 ms for /u/_*per*_, /u/_*aper*_, and /u/_*tone*_, respectively; *p *= n.s.). There were significant differences in all comparisons of the latency of the N1m elicited by the vowels /a/ and /u/ (*p *< 0.01 for /a/_*per *_vs. /u/_*per*_; *p *< 0.001 for /a/_*aper *_vs. /u/_*aper*_; *p *< 0.05 for /a/_*tone *_vs. /u/_*tone*_).

In the left hemisphere, the three variants of /u/ elicited the N1m at comparable latencies (126, 130, and 133 ms for /u/_*per*_, /u/_*aper*_, and /u/_*tone*_, respectively; *p *= n.s. in all comparisons), although the N1m tended to peak earlier as stimulus complexity was increased (/u/_*per *_vs. /u/_*tone*_, *p *= 0.07). Variations in the type of voice excitation had a marked effect on the latency of the N1m elicited by the vowel /a/: both the periodic and the aperiodic vowel elicited the N1m at a significantly longer latency than the two-tone complex (122, 123, and 114 ms for /a/_*per*_, /a/_*aper*_, and /a/_*tone*_, respectively; *p *< 0.05 for both /a/_*per *_and /a/_*aper *_vs. /a/_*tone*_). The 4-ms latency difference between the N1m responses to /a/_*per *_and /u/_*per *_was statistically non-significant, whereas the responses to /a/_*aper *_and /a/_*tone *_were faster than those to /u/_*aper *_and /u/_*tone *_(*p *< 0.05 for /a/_*aper *_vs. /u/_*aper*_; *p *< 0.001 for /a/_*tone *_vs. /u/_*tone*_).

**Figure 4:**
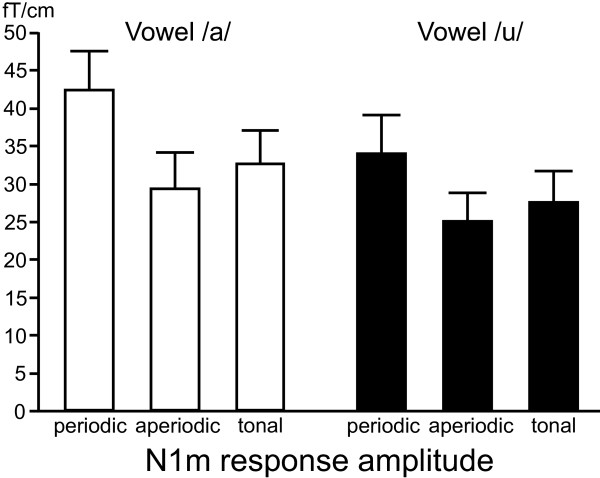
The grand-averaged amplitude of the N1m elicited by the vowels /a/ and /u/ with periodic, aperiodic, and tonal excitation (due to hemispheric symmetry, the left- and right-hemispheric data has been averaged). The vowels with periodic glottal excitation (/a/_per _& /u/_*per*_) elicited the most prominent N1m responses, and the amplitude difference between the two was statistically significant. In all cases, the vowels with aperiodic (/a/_*aper *_& /u/_*aper*_) and tonal (/a/_*tone *_& /u/_*tone*_) excitation resulted in N1m responses with significantly smaller amplitudes than did vowels with periodic excitation. Bars indicate standard error of the mean.

With regard to response amplitude, the right-hemispheric N1m responses were more prominent than the left-hemispheric ones (40 and 24 fT/cm; *F*(1,9) = 14.69, *p *< 0.01). In both hemispheres, the amplitude of the N1m varied according to both vowel category (*F*(1,9) = 5.54, *p *< 0.05; hemisphere-vowel-interaction (*F*(1,9) = 0.74, *p *= n.s.) and excitation type (*F*(2,18) = 17.35, *p *< 0.001; hemisphere-type of excitation – interaction (*F*(2,18) = 0.41, *p *= n.s.): As depicted in Fig. 4, the vowel /a/ elicited larger N1m responses than the vowel /u/ (35 and 29 fT/cm for /a/ and /u/, respectively, *p *< 0.05). Furthermore, the vowels with periodic excitation elicited larger-amplitude N1m responses (38 fT/cm) than the vowels with aperiodic (27 fT/cm) or tonal excitation (30 fT/cm; *p *< 0.001 for both periodic vs. aperiodic and periodic vs. tonal excitation). The vowels with aperiodic and tonal excitation, however, resulted in equally large N1m responses (*p *= n.s.).

**Figure 5:**
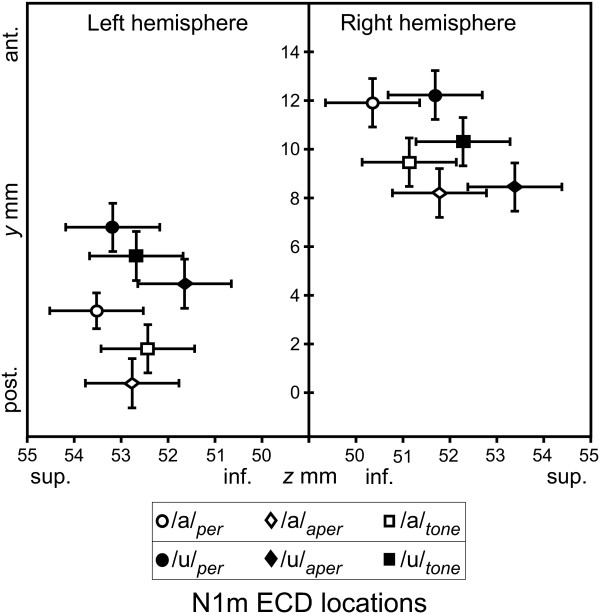
The ECD locations of the N1m responses in the anterior-posterior and superior-inferior dimensions. These were located in a restricted cortical area in both the left and the right hemisphere. The ECDs for the vowels with periodic (/a/_*per *_& /u/_*per*_) excitation were anterior to those for aperiodic (/a/_*aper *_& /u/_*aper*_) and tonal (/a/_*tone *_& /u/_*tone*_) excitation. Bars indicate standard error of the mean.

Corroborating previous observations [24-27], the sources of the N1m were confined to a restricted area in both hemispheres (displaying location shifts up to 7 mm), and the right-hemispheric ECD locations were more anterior than the left-hemispheric ones (Fig. 5). The N1m responses to stimuli with natural, periodic structure were anterior to those elicited by stimuli with impoverished stimulus structure. In both hemispheres, the ECDs for the periodic vowels (/a/_*per *_& /u/_*per*_) were roughly 3 mm anterior to those for the aperiodic vowels (/a/_*aper *_& /u/_*aper*_; *F*(2,4) = 15.98, *p *< 0.05 &* F*(2,6) = 6.62, *p *< 0.05 for the left and right hemispheres, respectively). The ECDs for the two-tone complexes (/a/_*tone *_& /u/_*tone*_) were located between those for the periodic and aperiodic vowels, differing statistically from neither. Also, there were no differences between the ECD locations either along the medio-lateral or the superior-inferior-dimension.

## Discussion

Here we studied the combined effects of phonation (i.e., voice excitation) and articulation (i.e., formant frequencies) on cortical activity elicited by vowels with carefully controlled acoustic properties. Brain activity elicited by natural, periodic speech sounds was contrasted with that elicited by the deficient harmonic structure of aperiodic speech sounds and two-tone complexes. Both the type of excitation of the vowels and their formant settings resulted in hemispheric asymmetries with regard to the latency behavior of the auditory N1m response, suggesting that the left and right auditory areas of the human brain employ different strategies for extracting information from speech signals. Further, given that the data revealing cortical asymmetries were derived in the passive recording condition, it appears that these extraction processes takes place without requiring, for example, top-down attentional engagement.

Firstly, we were able to establish that vowels comprising the periodic glottal excitation elicited distinctly different time courses of the auditory N1m in the left and right hemisphere: the vowel /a/ activated the right-hemispheric auditory cortex some 10 ms earlier than the vowel /u/, whereas both of these vowels activated the left-hemispheric auditory cortex at the same latency. This indicates that the right hemisphere treats differentially vowels with different formant settings and may therefore be involved in the processing of articulatory cues. The right-hemispheric 10-ms latency difference occurred regardless of the type of voice excitation and is compatible with previous observations [6,7,11,17,18] which have shown that the latency of the N1m is determined by the F1 and/or F2 frequency of the vowels, with the low-formant vowel /u/ eliciting a longer-latency N1m than the high-formant vowel /a/.

This latency effect of the N1m was complemented by modifications in the N1m amplitude according to both phonation and articulation. Phonation had a straightforward effect, with the natural periodic stimulation always resulting in more prominent brain activity than aperiodic or tonal stimulation. With regard to articulation, however, matters become more complicated because it appears that the N1m amplitude depends on both the locations of formant frequencies and the overall spectral distribution of the stimulus energy. Here, intensity matching was used to objectively normalize the overall energy (i.e., the energy integrated over all frequency components) to the same value for all the stimuli. This procedure is typically used in laboratory settings to ensure that different stimuli represent the same sound pressure level. Thus, using two clearly different articulatory settings, we were able to study the behavior of N1m evoked by speech sounds of equal phonation and overall energy but with different sound energy spectral distributions and established that the high-frequency periodic vowel /a/ elicits a larger-amplitude N1m than the periodic vowel /u/. The present data suggests that this could be attributed to differences in sound energy distributions: the periodic vowel /u/_*per*_, endowed with much lower frequency values of F1 and F2, has sound energy mainly at these frequencies, thus resulting in amplitude-diminished N1m response compared to the periodic vowel /a/_*per *_which has sound energy distributed across a wider range of high-energy harmonics. This interpretation gains further support if one considers the N1m amplitudes in Figure 4: the N1m amplitudes to the periodic vowel /u/ and the two-tone complexes, which have relatively similar distributions of spectral energy, are quite close to each other, whereas the large difference in N1m amplitudes elicited by the periodic vowel /a/ vs. the other five stimuli might reflect their large spectral discrepancy. Understanding the effects of sound energy distribution on the behavior of N1m obviously requires further experimentation and this could be done, for instance, by studying the processing of speech sounds representing the same phoneme, such as /a/, but excited by different shapes of the periodic glottal excitation. The present observations already indicate that the amplitude of the N1m is sensitive to the energy distribution of the stimulus which can be affected, importantly, both by changes in phonation and in articulation, and any violation in the natural structure of speech sounds is carried over to N1m amplitude and latency dynamics.

The present observations also suggest that the processing of periodic vowels with different spectral energy distributions results in latency changes in the right hemisphere whereas the left hemisphere responds to these vowels at an invariant latency. Therefore, we propose that the left-hemispheric constant-latency brain process in response to vowels with periodic glottal excitation is related to the ability to correctly categorize vowel identity irrespective of the considerable variations in their acoustic structure. This conclusion gains further support from a recent study [27] showing that the periodic vowel /a/ elicits the N1m at a constant latency regardless of whether the voice pitch is that of a male, a female, or a child. Here, the origin of speech-specific invariance in the left hemisphere is further narrowed down to the effects introduced by phonation, that is, the presence of the natural glottal excitation in stimulation: When the spectral comb structure provided by the periodic glottal excitation is replaced by an aperiodic one, the vowel with high-frequency F1 and F2 activate the auditory cortex at a significantly shorter latency than the vowel with low-frequency F1 and F2. When the spectral structure of the excitation is further impoverished, this latency difference becomes even more pronounced: the two-tone complex /a/_*tone *_activates the auditory cortex at a very short latency, characteristic of high-frequency tonal stimulation [11-13].

Finally, it appears that stimuli with a periodic spectral structure are processed in slightly different brain areas than stimuli with an aperiodic structure, there being shifts in the ECD locations in the anterior-posterior direction. Although the present observations provide corroborating evidence that the effect, despite being only of the order of 2–3 mm, is a reliable one [24], we are still lacking a proper explanation of the underlying neuronal mechanisms. Tentatively, one might suggest that stimuli with a natural harmonic structure evoke activity across larger neuronal populations than stimuli with an impoverished structure. Consequent changes in the centre of gravity of the activated cortical areas would show up as shifts in the ECD location as well as in larger response amplitudes for natural sounds. Alternatively, the more anterior activation for natural sounds might reflect the processing of speaker identity (present in the periodically excited vowels) which has been suggested to take place in anterior auditory areas (with posterior areas specializing in the processing of language content of stimulation [28,29]).

## Conclusion

The present study suggests that in human auditory cortex, categorization of speech sounds takes place irrespective of attentional engagement and is based on cues provided by both phonation (periodic glottal excitation) and articulation (the formants of voiced speech) which, consequently, lead to hemispheric asymmetries as indexed by the auditory N1m response. More specifically, the effect of the locations of the F1,F2 frequencies on the amplitude composition of the harmonics plays a major role in the categorical perception of vowels: The amplitude of the N1m in both hemispheres probably reflects the distribution of sound energy at different frequencies, and varies according to vowel category and the type of voice excitation. The latency variations of the right-hemispheric N1m appear to be attributable to the spectral energy distribution of the speech sound, while the invariant latency of the left-hemispheric N1m might be related to the ability of humans to categorize vowels irrespective of variations in pitch and loudness. The present study indicates that the simultaneous presence of the natural glottal excitation and formant frequencies is a prerequisite for the emergence of the speech-specific cortical activation as reflected in the auditory N1m response. Therefore, based on the above, we propose that speech-specificity should be understood as specificity to the acoustic structure of natural speech.

## Methods

### Subjects

Ten right-handed subjects (age 20 – 44 years, 6 females) participated in the study with informed consent. All the subjects reported being right-handed and having normal hearing. The experiment was approved by the Ethical Committee of the Helsinki University Central Hospital. During the experiment, the subjects, instructed not to pay attention to the auditory stimuli, were concentrating on reading a self-selected book or watching a silent video.

### Stimulus preparation and presentation

The stimuli (Fig. 1) were created by using the Semi-synthetic Speech Generation method [30]. Firstly, a natural glottal excitation (F0 = 115 Hz) was extracted from an utterance produced by a male speaker. By using this natural periodic glottal waveform as an input to an artificial vocal tract model, the vowels /a/_*per *_and /u/_*per *_of normal voice quality were synthesized. The lowest four formant frequencies of the vocal tract model were set at 670 Hz (F1), 1000 Hz (F2), 1950 Hz (F3) and 3440 Hz (F4) for /a/_*per *_and at 330 Hz (F1), 580 Hz (F2), 1900 Hz (F3) and 2900 Hz (F4) for /u/_*per*_. Secondly, the aperiodic counterparts of the vowels, /a/_*aper *_and /u/_*aper*_, were produced by replacing the glottal excitation with a noise sequence whose spectral envelope matched that of the glottal excitation. Thirdly, the two-tone complexes /a/_*tone *_and /u/_*tone *_were synthesized by exciting the vocal tract model with a composite of two sinusoidals. The frequencies and amplitudes of the tones were adjusted so that the spectrum of the synthesized tone complex matched the two strongest harmonics in the vicinity of F1 and F2 of the vowels /a/_*per *_and /u/_*per*_. This resulted in F1 and F2 values of 670 Hz & 1000 Hz for /a/_*tone *_and 330 Hz & 580 Hz for /u/_*tone*_, respectively. All the stimuli were smoothed during their onsets and offsets with a 5-ms Hanning-window. Finally, sound energy (computed as the squared sum of the digital time-domain signals) was equalized across the stimuli and the sound pressure level was adjusted for each subject by using the vowel /a/_*per *_as a reference stimulus resulting in a between-subject intensity range of 70–75 dB SPL(A). The 200-ms stimuli were delivered to the subject's ears through plastic tubes and ear pieces at an inter-stimulus interval of 800 ms. Each stimulus type was presented in its own sequence and the six sequences were presented in pseudorandom order counterbalanced across subjects. The presentation order was chosen randomly during each measurement and for each subject, and the order of stimulus presentation was controlled for to avoid possible short-term adaptation effects in the amplitude of the N1m.

### MEG data-acquisition and analysis

Cortical activation elicited by the stimuli was registered by using a 306-channel whole-head MEG measurement device (Elekta Neuromag Oy, Finland) in a magnetically shielded room. At the beginning of each stimulus sequence, the head position with respect to the sensor array was determined by using head position indicator coils attached to the subjects scalp, with the locations of the coils with respect to the left and right preauricular points and the nasion having been determined prior to the measurement. In order to cancel out the cortical activity not time-locked to stimulus presentation (e.g., activity related to muscle artefact, eye-movements caused by reading or watching the video), for each stimulus, 150 evoked responses were averaged over a period of 700 ms including a 100-ms pre-stimulus baseline, and passband-filtered at 1–30 Hz. Epochs exceeding 3000 fT/cm were excluded online, and electrodes monitoring horizontal and vertical eye movements were used in removing artefacts (>150 μV) online.

The auditory N1m, defined as the response maximum in the registered waveform at around 100 ms, was studied for effects in amplitude and latency. In each hemisphere and for each subject, response latency was determined from the pair of planar gradiometers exhibiting N1m response maxima (which was the same for all stimulus types) for all the waveforms elicited by the different stimulus types. Response amplitude was defined as the average of the field gradient vector sums from six pairs of planar gradiometers displaying maximum N1m responses. Source localization was done by using unrestricted single equivalent current dipoles (ECDs). The ECDs were fitted to a single time point defined as the moment of the N1m reaching its peak amplitude in the averaged waveform of all the 66 sensors located above the left or right temporal brain areas. The ECD locations were estimated in a three-dimensional coordinate system defined by the *x*-axis passing through the preauricular points (positive to the right), the *y*-axis passing through the nasion, and the *z*-axis as the vector cross-product of the *x *and *y *unit vectors. Statistical analyses were performed by using repeated measures ANOVA (2 hemispheres × 2 vowels × 3 excitation types for the response waveforms; 2 vowels × 3 types of excitation separately in the right and the left hemispheres for the ECD locations) and Newman-Keuls *post hoc *-tests when appropriate.

## Authors' contributions

HT, AMM and PA designed the experimental setup of the study, and PA prepared the auditory stimuli. AMM and VM acquired the data. AMM performed the data & statistical analyses. All authors participated in the writing process, and have approved the final version of the manuscript.
